# Atmospheric Aerosol Neurotoxicity and Parkinson's Disease: Mechanisms and Perspectives

**DOI:** 10.1002/jbt.71089

**Published:** 2026-08-26

**Authors:** Yitian Gao, Minxiang Dong, Chenyang Duan, Xiaohan Zheng, Liming Qu

**Affiliations:** ^1^ College of Geographical Sciences, Faculty of Geographical Science and Engineering Henan University Kaifeng China; ^2^ The Second Affiliated Hospital of Inner Mongolia Medical University Inner Mongolia Medical University Hohhot China; ^3^ Huaihe Hospital of Henan University Henan University Kaifeng China

**Keywords:** atmospheric aerosols, neurotoxicity, oxidative stress, Parkinson's disease, α‐synuclein

## Abstract

Parkinson's disease (PD) is a common neurodegenerative disease threatening the health of the middle‐aged and elderly population, triggered by the combined effects of genetic susceptibility, environmental exposure and nervous system aging, among which atmospheric aerosol exposure is a key risk factor for sporadic PD. As a complex heterogeneous mixture, atmospheric aerosols contain neurotoxic components such as metal particles, persistent organic pollutants (POPs), and nano/ultrafine particles, which appear to target midbrain substantia nigra dopaminergic neurons through pathways including direct entry into the brain via the olfactory pathway, penetration across the lung‐blood‐brain barrier, and inflammation‐mediated alterations in barrier permeability. The core molecular mechanisms that have been proposed to link aerosol exposure to sporadic PD include oxidative stress and mitochondrial dysfunction, neuroinflammation and aberrant microglial activation, disrupted protein homeostasis and abnormal aggregation of α‐synuclein (α‐syn), as well as altered epigenetic regulation. Moreover, neurotoxic effects may be amplified via the positive feedback loop of oxidative stress‐neuroinflammation, the vicious cycle of mitophagy defects and protein aggregation, and the “multiple‐hit hypothesis”. This review summarizes the toxic components of aerosols, their brain‐entry routes and pathogenic mechanisms, outlines the application status of emerging technologies such as in vitro cell models and in vivo animal models, and sorts out intervention strategies including pharmacological therapy and epigenetic regulation. It also analyzes the current challenges in research on mechanisms, models, population studies and intervention strategies, and points out that future research should focus on dissecting the synergistic mechanisms of multiple components and optimizing research models, so as to provide theoretical support for exploring potential strategies for precise prevention, control and treatment of PD.

## Graphical abstract

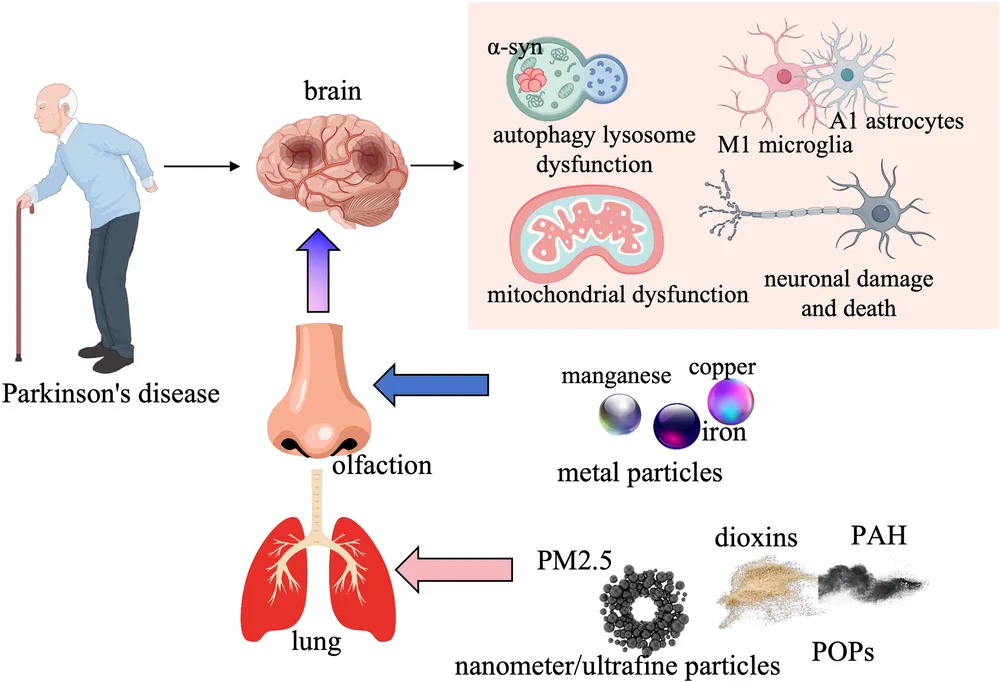

## 1 Introduction

Parkinson's disease (PD) ranks as the second most prevalent progressive neurodegenerative disorder worldwide, with robust epidemiological data demonstrating steep age‐dependent prevalence that peaks among adults aged 80 years and older [1, 2]. Large‐scale Global Burden of Disease (GBD) meta‐analyses consistently document higher disease prevalence in male populations, a disparity attributed to combined gender‐specific differences in occupational neurotoxicant exposure and endogenous estrogen‐mediated neuroprotective hormone profiles [2]. The fastest growing PD disease burden is currently observed within middle‐income nations undergoing rapid industrialization and urban expansion; this epidemiological trend is largely driven by anthropogenic environmental pollution and sustained population aging across these regions [3]. Genetically determined familial PD accounts for only approximately 10% of all clinical PD cases, while the remaining 90% are classified as sporadic PD, where cumulative environmental toxicant exposure acts as a central pathogenic driver interacting with individual genetic susceptibility [4, 5]. Multiple cohort and case–control epidemiological investigations have validated significant PD risk elevation linked to chronic exposure to agrochemical toxicants such as paraquat and chlorpyrifos; subsequent systematic reviews further expand this association to widespread air pollutants and heavy metal contamination [6, 7].

**Figure 1:**
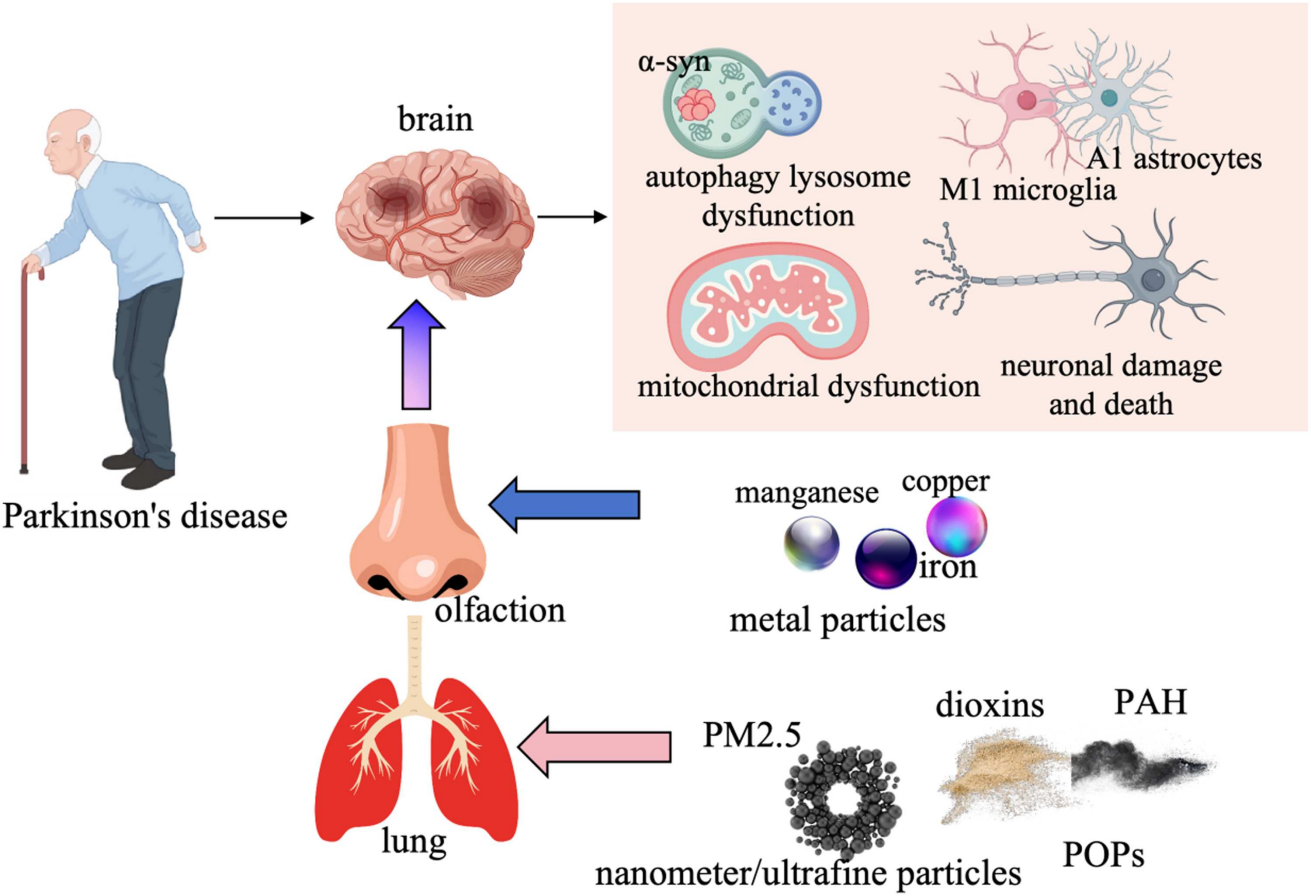
Schematic overview of the pathways and pathological mechanisms connecting atmospheric aerosol exposure to PD. Atmospheric aerosols, including PM2.5 and nanometer/ultrafine particles loaded with metal particles, persistent organic pollutants (POPs), polycyclic aromatic hydrocarbons (PAHs) and dioxins, enter the body predominantly via pulmonary inhalation. Nano/ultrafine particles can also directly access the brain through the olfactory route. Within the central nervous system, aerosol toxicants appear to trigger mitochondrial dysfunction, impaired autophagy‐lysosome function and α‐synuclein pathology, accompanied by pro‐inflammatory activation of M1 microglia and A1 astrocytes. These pathological processes collectively may lead to neuronal injury and loss, which is linked to the onset and progression of PD.

Among all air pollution hazards, atmospheric aerosol exposure represents a globally pervasive environmental threat whose causal correlation with sporadic PD has attracted rapidly expanding research attention over the past decade [8]. A large body of recent meta‐analyses and long‐term population cohorts provides solid epidemiological evidence that chronic PM2.5 exposure significantly increases the incidence risk of sporadic PD with a clear dose–response relationship; however, available human longitudinal data are insufficient to robustly confirm that PM2.5 exposure accelerates disease progression in already diagnosed PD patients, with relevant supportive findings mostly originating from in vitro and animal toxicological experiments [9, 10]. Long‐term low‐concentration aerosol toxicant exposure induces silent, progressive neuronal damage that manifests clinically decades after initial exposure; such subclinical neural injury synergizes with aging and inherent genetic vulnerability to jointly initiate and accelerate PD pathological progression [7, 11]. Despite accumulating correlational population evidence linking aerosols to PD onset, the integrated neurotoxic pathways through which multicomponent atmospheric pollutants target midbrain dopaminergic neurons remain incompletely characterized, with critical mechanistic and translational knowledge gaps unresolved. This review systematically delineates neurotoxic aerosol constituents, their multiple brain translocation routes, and core molecular pathological cascades; it further evaluates strengths and limitations of prevailing in vitro, in vivo and human population research models, summarizes candidate pharmacological and public health intervention approaches, and discusses outstanding challenges across mechanistic dissection, experimental design, population research and therapeutic translation. The overarching objective of this work is to deliver integrated theoretical evidence supporting the development of precise prevention, early screening and targeted therapeutic strategies for aerosol‐associated sporadic PD (Figure 1).

## 2 Neurotoxic Components of Atmospheric Aerosols and Their Brain Entry Pathways

### 2.1 Key Toxic Components

#### 2.1.1 Metal Particles (Fe, Mn, Cu, etc.) and Their Redox Activity

**Figure 2:**
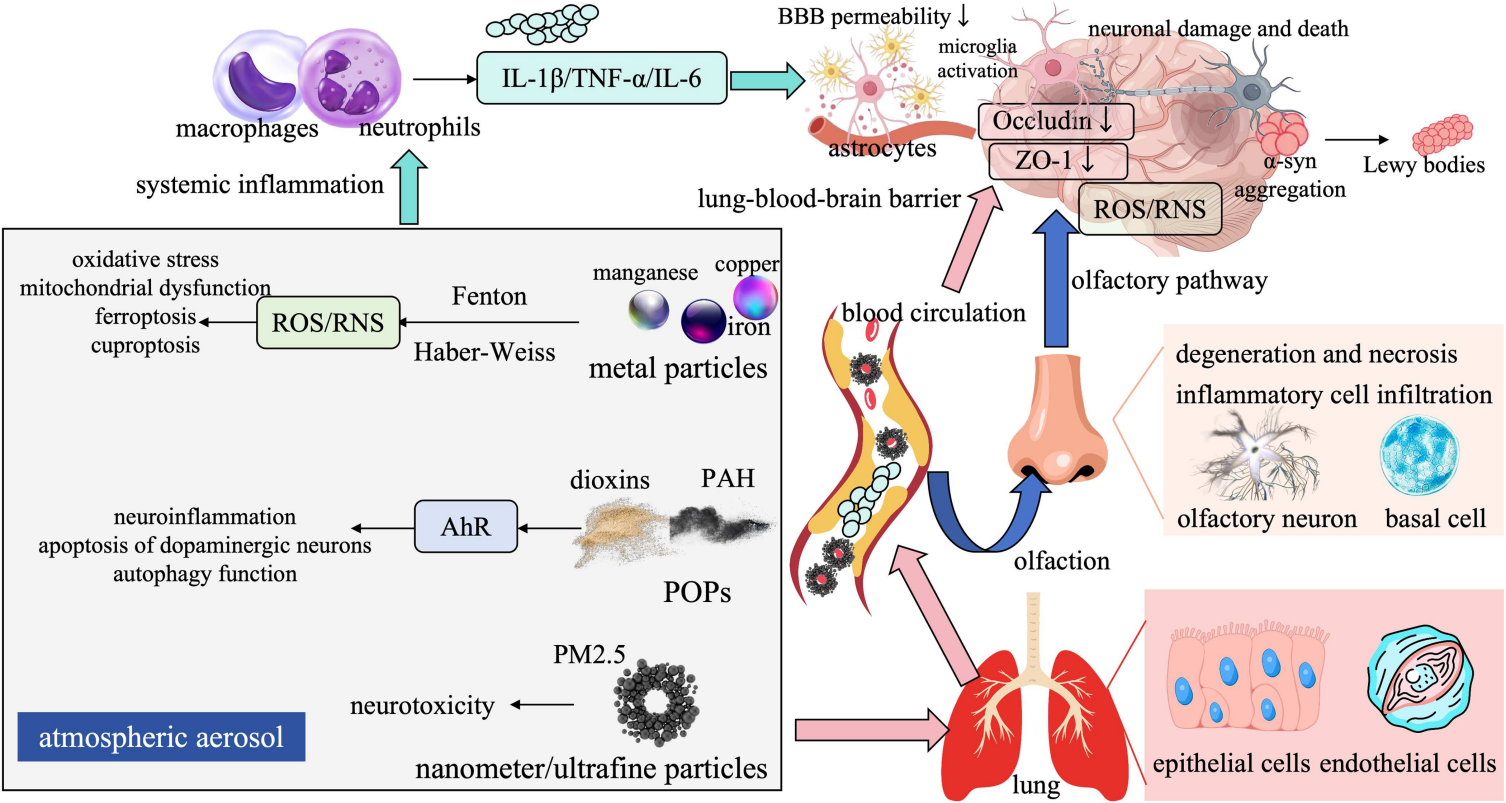
Neurotoxic components and brain entry mechanisms of PD induced by atmospheric aerosols. Metallic particles in atmospheric aerosols may generate ROS/RNS via Fenton and Haber‐Weiss reactions, which appears to trigger oxidative stress and mitochondrial damage, and is hypothesized to induce ferroptosis and cuprotosis that harm dopaminergic neurons in experimental models. POPs such as PAHs and dioxins may elicit neuroinflammation, neuronal apoptosis, and autophagy disturbance by activating the AhR pathway in vitro. Nano/ultrafine particles may synergistically amplify toxic effects through their high adsorptivity and penetrability. The brain entry pathways include: direct transport to the olfactory bulb and brain nuclei via the olfactory pathway, which may lead to early olfactory impairment; nanoparticles and soluble components cross the alveoli into the bloodstream, may disrupt tight junction proteins, and increase BBB permeability. Meanwhile, aerosols may activate peripheral inflammatory cells to release inflammatory factors, supporting systemic inflammation, which further may compromise the BBB, activate microglia, and amplify central inflammatory responses. Ultimately, these toxic processes collectively may contribute to necrosis of dopaminergic neurons and aggregation of α‐syn to form Lewy bodies, which is associated with the occurrence and progression of PD.

Metal particles are important neurotoxic components in atmospheric aerosols, mainly derived from industrial activities (metallurgy, electroplating), fossil fuel combustion, traffic emissions, soil dust, and other sources. Among them, transition metal particles such as iron, manganese, and copper are the most common and exert well‑defined neurotoxicity [12, 13]. The mechanistic links between these metals and dopaminergic neuronal injury are supported by three tiers of evidence with distinct translational value, which are integrated without clear differentiation in the original text and are separated here for clarity. In vitro cell assays using primary midbrain dopaminergic neurons and SH‐SY5Y cell lines confirm that these metal particles possess strong redox activity; upon cellular uptake, they catalyze robust generation of reactive oxygen species (ROS) and reactive nitrogen species (RNS) via Fenton reactions and Haber–Weiss reactions, disrupting intracellular redox homeostasis and triggering oxidative damage [14] (Figure 2, Table 1). In particular, copper ions directly bind intracellular α‑synuclein (α‑syn), driving its conformational rearrangement, oligomerization and fibrilization to promote Lewy body formation, while simultaneously worsening oxidative stress and mitochondrial injury to amplify neurotoxicity in precellular models [15, 16]. Despite clearly illustrating direct metal‐neuron interactions, these in vitro systems fail to replicate intact respiratory absorption, systemic circulation and blood‐brain barrier (BBB) transport pathways that exist in living organisms. In vivo rodent exposure models, including chronic intranasal instillation and whole‐body inhalation regimens, further demonstrate that long‐term manganese exposure leads to metal deposition within the midbrain substantia nigra and selectively impairs dopaminergic neurons through manganese‐dependent oxidative stress and mitochondrial dysfunction [17].

**Table 1:** Major atmospheric aerosol components linked to PD: sources, targets, mechanisms, evidence, and limitations.

Aerosol toxic component	Primary emission sources	Key biological targets in CNS	Core neurotoxic molecular mechanisms	Supporting evidence type	Major research limitations
Transition metal particles (Fe, Mn, Cu) [12, 13, 14, 15]	Metallurgy, vehicle exhaust, industrial fumes, soil dust, coal combustion	Substantia nigra dopaminergic neurons, mitochondria	1. Fenton/Haber‐Weiss reaction overproducing ROS/RNS	In vitro cell assays, intranasal animal exposure, occupational cohort epidemiology	Mixed metal co‐exposure synergistic toxicity rarely quantified; low‐dose long‐term cumulative damage hard to simulate; human in vivo metal distribution data scarce
			2. Inhibit mitochondrial Complex I, trigger energy deficit		
			3. Bind α‐syn to may facilitate oligomer formation		
			4. Induce ferroptosis/cuproptosis5. Epigenetic suppression of PINK1/Parkin		
PAHs [18, 19, 20]	Fossil fuel burning, traffic, industrial waste, cooking fumes	Dopaminergic neurons, microglia, astrocytes	1. Metabolic activation generates oxidative intermediates	Primary neuron culture, whole‐body inhalation mice, population biomonitoring	Complex mixture co‐pollutant interference; difficult to separate PAH‐specific effects from other POPs; individual internal exposure dose hard to quantify
			2. Activate AhR pathway to trigger neuroinflammation		
			3. Hypermethylate BDNF promoter to reduce neurotrophic support		
			4. Impair lysosomal autophagy flux		
Dioxins [21, 22, 23]	Industrial chemical waste, waste incineration, pesticide residues	Microglia, dopaminergic neurons	1. Persistent AhR‐mediated chronic neuroinflammation	In vitro glia‐neuron co‐culture, chronic rodent exposure	Low environmental concentration; long latency of neuronal injury; limited large‐scale human cohort data
			2. Suppress dopamine synthase activity		
			3. Accelerate α‐syn intercellular propagation		
Organophosphorus pesticides (Chlorpyrifos) [21, 22, 23]	Agricultural spraying, atmospheric drift, dust resuspension	Autophagy‐lysosome system, dopaminergic neurons	1. Block autophagic flux, impair mitophagy	Transgenic PD mouse exposure, agricultural case–control studies	Confounding lifestyle/occupational factors in human studies; multipesticide combined exposure unclear
			2. Elevate α‐syn intracellular accumulation		
			3. Induce NLRP3 inflammasome activation		
Nano/ultrafine particles (≤ 0.1 μm, soot nanoparticles) [24, 25, 26, 27]	Diesel exhaust, industrial nanomaterial production, coal soot	Olfactory epithelium, BBB endothelial cells, substantia nigra	1. High adsorption capacity to carry metals/POPs into brain	Nose‐only inhalation models, intranasal instillation, brain organoid tests	Real‐world mixed particle composition hard to replicate in lab; particle size/charge/shape joint toxicity insufficiently studied
			2. Direct olfactory bypass entry to CNS		
			3. Disrupt tight junctions (ZO‐1/Occludin) to increase BBB permeability		
			4. Induce microglial M1 polarization and ferroptosis		

Animal models faithfully recapitulate the human olfactory and pulmonary routes of aerosol entry into the central nervous system, yet interspecies differences in dopamine metabolism, olfactory neural circuits and metal clearance capacity restrict direct extrapolation of rodent toxicological outcomes to human sporadic PD [28, 29]. Human epidemiological evidence from occupational cohorts of metallurgy and welding workers reveals that chronic manganese inhalation correlates with elevated PD risk, and community population biomonitoring identifies positive correlations between peripheral blood metal levels and systemic oxidative stress markers [30, 31]. However, population‐level observations only establish correlational associations rather than definitive causal relationships, as confounding factors including co‐occurring persistent organic pollutants (POPs) co‐exposure, genetic susceptibility and variable lifestyle cannot be fully adjusted to isolate metal particles as the direct pathological trigger. In addition, these metal particles can adsorb other neurotoxic substances and synergistically enhance the overall neurotoxicity of atmospheric aerosols.

#### 2.1.2 POPs (Polycyclic Aromatic Hydrocarbons (PAHs), Dioxins)

POPs are a class of organic compounds characterized by high toxicity, resistance to degradation, and strong bioaccumulation. They mainly originate from fossil fuel combustion, industrial production, pesticide application, and other sources. Common POPs associated with PD include PAHs, dioxins, organochlorine pesticides, and others. These pollutants are widely distributed in the environment via adsorption onto atmospheric aerosols. Long‐term exposure leads to their accumulation in adipose tissue, the nervous system, and other tissues, where they may exert persistent neurotoxic effects in preclinical models [18, 19]. PAHs are the most prevalent POPs in atmospheric aerosols. Their metabolites, such as benzo[a]pyrene diol epoxide, exhibit strong carcinogenic and neurotoxic potential in vitro, appear to target and harm dopaminergic neurons by driving oxidative stress, DNA damage, and disturbed protein homeostasis [20]. As a highly toxic pollutant, dioxins may activate the aryl hydrocarbon receptor (AhR) signaling pathway, which has been linked to neuroinflammation, suppressed activity of dopamine‐synthesizing enzymes, reduced dopamine secretion, and elevated dopaminergic neuron apoptosis in laboratory assays [21]. In addition, chlorpyrifos, a widely used organophosphorus pesticide, can form inhalable exposures via atmospheric aerosols. Long‐term exposure is associated with a nearly twofold increased risk of PD in epidemiological studies, and its neurotoxicity is proposed to be mainly mediated by impaired neuronal autophagy and the accumulation of abnormal proteins in experimental systems [22, 23].

#### 2.1.3 Physical Properties of Nanoparticles/Ultrafine Particles

Nanoparticles and ultrafine particles (particle size ≤ 0.1 μm) represent one of the most potentially neurotoxic components in atmospheric aerosols. They are mainly derived from fossil fuel combustion, industrial nanomaterial production, traffic emissions, and other sources. Their neurotoxic potential appears to correlate with their unique physical properties. Compared with coarse particles, nanoparticles and ultrafine particles possess a large specific surface area, high surface reactivity, and strong penetration ability. They can adsorb large amounts of neurotoxic substances such as metal particles and POPs, which may substantially amplify overall toxic effects in laboratory models [24]. Due to their extremely small size, they can readily penetrate the respiratory mucosal barrier and alveolar barrier, enter the systemic circulation, and subsequently cross the BBB into the brain in animal experiments. They can also directly enter the brain via the nasal olfactory epithelium, bypassing the BBB and may hasten the emergence of neurotoxic alterations in preclinical systems [25]. Furthermore, physical properties such as surface charge and shape appear to modulate neurotoxicity. Positively charged nanoparticles tend to bind more easily to cell membranes, which may cause membrane damage, while irregularly shaped nanoparticles can elicit more severe mechanical injury to cultured cells [26]. Soot nanoparticles, a major component of PM2.5, have been shown to trigger ferroptosis in dopaminergic neurons in vitro and in vivo, a process that has been proposed as a novel mechanism potentially contributing to the development and progression of PD, further highlighting the neurotoxic potential of nanoparticles and ultrafine particles [27].

### 2.2 Transport Mechanisms From Exposure Sites to the Brain

#### 2.2.1 Direct Brain Entry via the Olfactory Pathway

The olfactory pathway serves as a critical route for atmospheric aerosols (especially nanoparticles/ultrafine particles and lipophilic pollutants) to directly enter the brain, and is also one of the early targets of pathological alterations in PD. Patients with PD often exhibit hyposmia at an early stage, which is closely associated with pathological injury in the olfactory pathway, and atmospheric aerosol exposure has been hypothesized as a key factor contributing to this phenomenon [32]. Toxic components in atmospheric aerosols, including nanoparticles/ultrafine particles and polycyclic aromatic hydrocarbons, can enter the nasal cavity with inhaled air, contact the nasal olfactory epithelium, and be captured by cilia of olfactory neurons in animal models (Figure 2, Table 1). These components may reach the brain via two routes: first, through axonal transport in olfactory neurons, directly reaching the brain along the olfactory bulb–olfactory tract–limbic system pathway, which appears to target the olfactory bulb, hippocampus, substantia nigra and other regions and may drive local neural damage in rodents [33, 34]; second, entering the lamina propria of the olfactory mucosa through basal cells of the olfactory epithelium, and then reaching the brain indirectly via the lymphatic or blood circulation, although the transport efficiency of this pathway is much lower than direct axonal transport [33, 34]. Studies have shown that in mice chronically exposed to atmospheric aerosols, massive particle accumulation can be detected in the olfactory bulb, accompanied by degeneration and necrosis of olfactory neurons and inflammatory cell infiltration [35], further supporting the critical role of the olfactory pathway in mediating brain entry of aerosol toxicants in preclinical systems. Such damage may emerge prior to the appearance of typical motor symptoms of PD.

#### 2.2.2 Penetration and Disruption of the Lung‐Blood‐Brain Barrier

Penetration through the lung‐blood‐brain barrier is another major pathway for atmospheric aerosols to enter the brain, mainly applicable to particles capable of crossing the alveolar barrier (e.g., nanoparticles/ultrafine particles) and soluble toxic components (e.g., metal ions, soluble organic pollutants). After inhalation into the respiratory tract, fine particles with a diameter ≤ 2.5 μm can reach the alveoli, among which nanoparticles/ultrafine particles may directly cross alveolar epithelial cells and capillary endothelial cells to enter the systemic circulation in animal assays [36]. Soluble toxicants (e.g., iron, manganese ions, metabolites of polycyclic aromatic hydrocarbons) can enter the circulation via passive diffusion or active transport across alveolar epithelial cells [37]. Toxic components in the bloodstream must cross the BBB to reach the brain and may exert neurotoxic effects (Figure 2, Table 1). However, nanoparticles/ultrafine particles in atmospheric aerosols appear to cross the BBB through multiple mechanisms in preclinical models: first, via endocytosis by endothelial cells, followed by transcytosis into the brain parenchyma; second, by compromising BBB integrity and increasing barrier permeability, which may permit toxicants to enter the brain [38, 39]. For instance, PM2.5 exposure is associated with reduced expression of tight junction proteins (e.g., Occludin, zonula occludens‐1 [ZO‐1]) in BBB endothelial cells in vitro, which may disrupt tight junction structures, raise BBB permeability, and allow more toxic components to enter the brain and selectively may impair dopaminergic neurons [40].

#### 2.2.3 Systemic Inflammation and Altered Barrier Permeability

Atmospheric aerosol exposure may trigger systemic inflammation in human and animal studies, which in turn could facilitate brain entry of toxic components by increasing barrier permeability. This represents an indirect brain entry route and a key mechanism proposed to amplify aerosol‐linked neurotoxicity. Upon entering the respiratory tract or bloodstream, toxic components in atmospheric aerosols (e.g., metal particles, polycyclic aromatic hydrocarbons) may activate the immune system, inducing recruitment and activation of inflammatory cells such as macrophages and neutrophils, which release pro‐inflammatory cytokines including interleukin‐1β (IL‐1β), tumor necrosis factor‐α (TNF‐α), and interleukin‐6 (IL‐6), thereby supporting systemic inflammatory responses [41]. These cytokines can reach the brain through the circulation, act on endothelial cells and astrocytes of the BBB, reduce the expression of endothelial tight junction proteins, may disrupt BBB integrity, and increase barrier permeability [42]. Meanwhile, inflammatory cytokines may activate microglia in the brain, driving central nervous system inflammation and further potentiating neural injury in preclinical systems [43]. In addition, systemic inflammation may disturb the body's redox balance, facilitate ROS production, and indirectly harm the BBB and brain parenchymal cells, creating favorable conditions for brain entry of aerosol toxicants [44] (Figure 2, Table 1). Epidemiological studies have revealed that individuals chronically exposed to atmospheric aerosols exhibit significantly elevated circulating levels of inflammatory cytokines, which are positively correlated with an increased risk of PD, suggesting that systemic inflammation may serve as a vital intermediate pathway linking aerosol exposure to PD‐related pathological changes [45].

## 3 Core Molecular Mechanisms of Neurotoxicity and Parkinson's Disease‐Related Pathology Induced by Atmospheric Aerosols

### 3.1 Oxidative Stress and Mitochondrial Dysfunction

#### 3.1.1 Explosive Generation of ROS/RNS and Collapse of Antioxidant Defense

**Figure 3:**
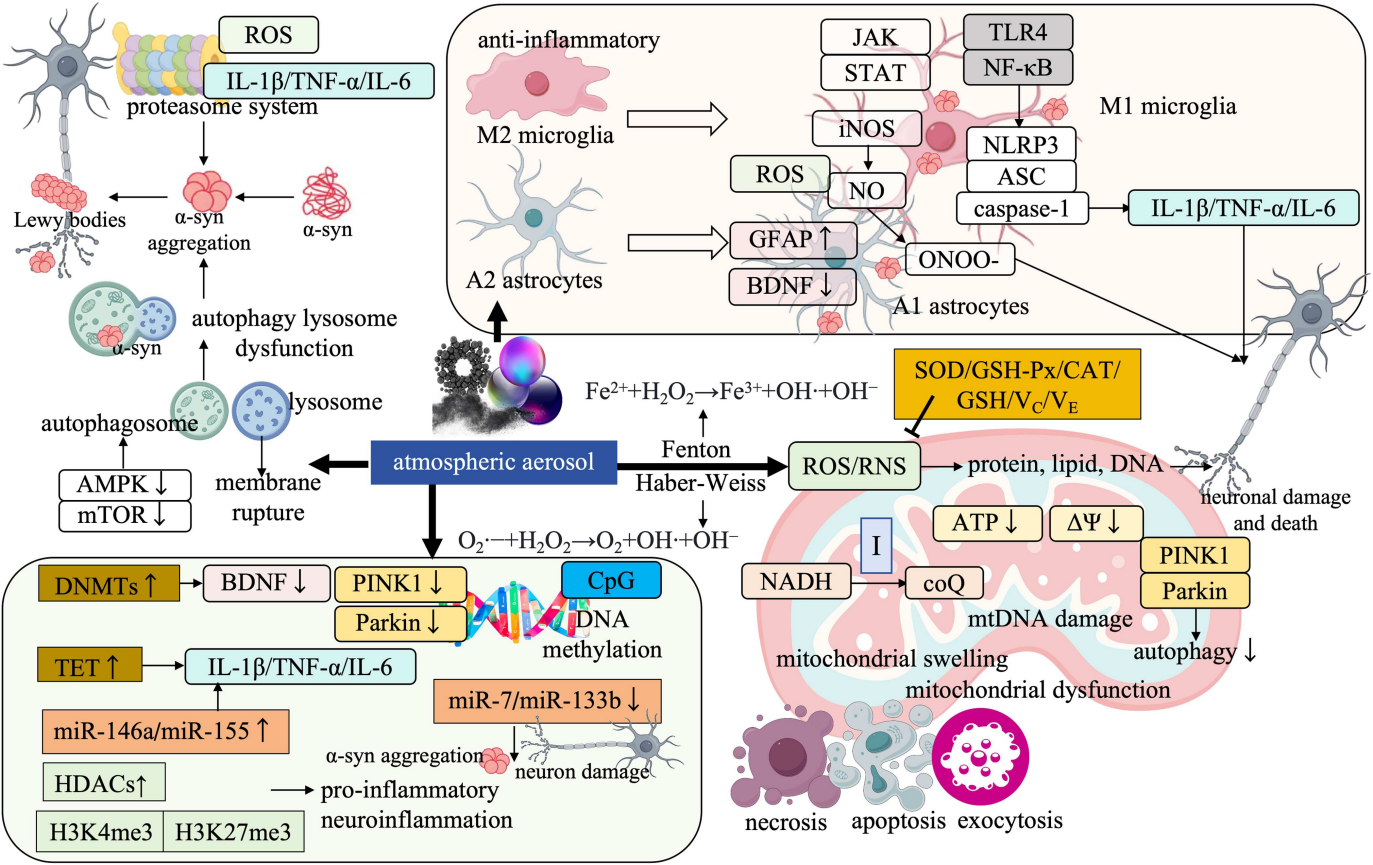
Core molecular mechanisms of dopaminergic neuron injury and PD‐related pathology induced by atmospheric aerosols. Atmospheric aerosols induce oxidative stress, neuroinflammation, protein homeostasis imbalance, and epigenetic regulation disorders through the synergistic effect of multiple pathways, ultimately leading to dopaminergic neuron injury and the pathological process of PD. In terms of oxidative stress and mitochondrial dysfunction, toxic components of aerosols (such as metal particles, nanoparticles, polycyclic aromatic hydrocarbons) can induce the massive generation of ROS/RNS through Fenton/Haber‐Weiss reactions and activation of NOX. Meanwhile, they inhibit antioxidant enzymes such as SOD, GSH‐Px, and CAT, as well as non‐enzymatic antioxidants such as GSH, vitamin C, and vitamin E, resulting in oxidative stress damage and oxidative modification of proteins, lipids, and DNA. Additionally, aerosols can directly inhibit the activity of mitochondrial complex I, block electron transport, leading to decreased ATP production, reduced membrane potential (ΔΨ), and mtDNA damage. Electron leakage further exacerbates ROS generation, forming a vicious cycle. Moreover, ROS can further inhibit PINK1/Parkin‐mediated mitophagy, impair mitochondrial clearance function, and aggravate mitochondrial swelling and dopaminergic neuron apoptosis. In terms of neuroinflammation and glial cell activation, ROS and mitochondrial damage may stimulate the NLRP3‐ASC‐caspase‐1 complex in microglia, cleave and generate mature IL‐1β and IL‐18, and simultaneously may stimulate the NF‐κB signaling pathway to promote the release of pro‐inflammatory factors such as TNF‐α and IL‐6, may eliciting neuroinflammation. Aerosols can also induce the polarization of microglia to the pro‐inflammatory phenotype (M1 type) through TLR4/NF‐κB and JAK/STAT signaling pathways, which secrete iNOS, NO, and pro‐inflammatory factors to directly damage dopaminergic neurons. Peroxynitrite (ONOO‐) generated by NO and ROS further amplifies the toxicity. In addition, aerosols can induce the activation of astrocytes to the A1 type, up‐regulate the expression of GFAP, reduce the secretion of BDNF, form glial scars, and promote the release of pro‐inflammatory factors, thereby weakening the nutritional support and protection for dopaminergic neurons. In terms of protein homeostasis imbalance and abnormal aggregation of α‐syn, aerosols can directly induce the conformational change of α‐syn from soluble α‐helix to insoluble β‐sheet, promote its oligomerization and fibrosis, and form Lewy bodies. Oxidative stress and inflammation further exacerbate this process. Meanwhile, ROS and pro‐inflammatory factors inhibit the activity of the proteasome system, may suppress autophagic flux initiation through the AMPK/mTOR signaling pathway, damage the integrity of the lysosomal membrane, lead to blocked autophagic flux, and result in the accumulation of abnormal α‐syn in cells due to ineffective clearance. Aerosols can also induce cell necrosis/apoptosis or may stimulate exocytotic pathways, promoting the release of abnormal α‐syn into the extracellular environment. The released abnormal α‐syn is then taken up by surrounding healthy dopaminergic neurons and glial cells, inducing the aggregation of endogenous α‐syn and leading to pathological spread. In terms of epigenetic regulation disorders, aerosols can up‐regulate the activity of DNMTs, leading to increased methylation and decreased expression of the promoters of protective genes related to PD (such as BDNF, PINK1, and Parkin). Meanwhile, they inhibit TET demethylases, resulting in decreased methylation and increased expression of pro‐inflammatory genes (such as IL‐1β and TNF‐α). Aerosols can also down‐regulate miR‐7 and miR‐133b, relieving the inhibition on α‐syn and affecting dopaminergic neuron survival, and up‐regulate miR‐146a and miR‐155 to enhance neuroinflammatory responses. In addition, aerosols can may stimulate histone deacetylases (HDACs), reduce histone acetylation levels, and inhibit protective genes such as BDNF. At the same time, they may stimulate histone methyltransferases, leading to abnormal modifications of H3K4me3 and H3K27me3, regulating the expression of inflammation‐related genes and maintaining persistent neuroinflammation.

Oxidative stress is one of the core mechanisms proposed to explain dopaminergic neuronal damage linked to atmospheric aerosol exposure, and also an important pathological feature observed in PD patients. Multiple toxic components in atmospheric aerosols (e.g., metal particles, nano/ultrafine particles, polycyclic aromatic hydrocarbons) drive massive ROS and RNS production, while suppressing endogenous antioxidant defense systems, and the supporting evidence for this cascade can be divided into in vitro, in vivo and human population data sets with differing translational strengths, which were previously mixed without clear distinction (Figure 3). In vitro experimental systems including primary dopaminergic neuron cultures and human midbrain organoids show that transition metals catalyze hydrogen peroxide (H_2_O_2_) conversion into highly reactive hydroxyl radicals (·OH) through Fenton and Haber–Weiss reactions, broadly damaging intracellular proteins, lipids and DNA [46, 47]. Nano/ultrafine particles activate intracellular nicotinamide adenine dinucleotide phosphate (NADPH) oxidase (NOX) to boost superoxide anion generation, and surface‐adsorbed toxicants further amplify ROS accumulation; organic pollutants such as PAHs generate reactive metabolic intermediates to stimulate oxidative bursts and simultaneously inhibit antioxidant enzyme activity [48, 49]. These simplified cellular platforms enable precise dissection of subcellular toxic events but lack systemic pulmonary absorption, circulating inflammatory signaling and intact BBB regulation found in mammals. Long‐term whole‐body inhalation mouse models mimic lifelong human ambient aerosol exposure, and exposed animals exhibit significant suppression of superoxide dismutase (SOD) and glutathione peroxidase (GSH‐Px) activity alongside depleted intracellular glutathione (GSH) within substantia nigra dopaminergic neurons, leading to unregulated ROS accumulation, lipid peroxidation and neuronal ferroptosis [50, 51].

Animal models reconstruct the complete lung‐to‐brain exposure cascade, yet most laboratory exposure concentrations exceed typical low‐dose chronic human environmental exposure, creating uncertainty for direct clinical translation [52]. Human epidemiological investigations of residents in high‐pollution regions identify consistent elevation of circulating oxidative stress biomarkers in populations with long‐term PM2.5 exposure, and multiple cohort studies confirm positive dose‐response relationships between aerosol exposure intensity and PD incidence risk [53, 54]. Nevertheless, human peripheral biomarker measurements only reflect systemic peripheral damage and cannot directly visualize mitochondrial and neuronal lesions within the human midbrain substantia nigra, requiring complementary toxicological models to validate causal molecular pathways. The body's antioxidant defense system mainly consists of enzymatic antioxidants [SOD, GSH‐Px, catalase (CAT)] and non‐enzymatic antioxidants [GSH, vitamin C (V_C_), vitamin E (V_E_)], which eliminate excessive ROS/RNS to maintain redox homeostasis. However, long‐term exposure to atmospheric aerosols causes ROS/RNS production to exceed the scavenging capacity of the antioxidant system, suppresses antioxidant enzyme activity, and reduces non‐enzymatic antioxidant levels, resulting in the collapse of antioxidant defense [55, 56]. For instance, PM2.5 exposure significantly decreases the activities of SOD and GSH‐Px and reduces GSH content in dopaminergic neurons, leading to massive ROS accumulation [57]. This further triggers cell membrane lipid peroxidation, protein oxidative modification, and DNA damage, ultimately causing degeneration and necrosis of dopaminergic neurons. In addition, oxidative stress can induce ferroptosis in dopaminergic neurons and accelerate PD‐related pathological progression [58].

#### 3.1.2 Inhibition of Mitochondrial Complex I and Energy Metabolism Crisis

Mitochondria are the core site of intracellular energy metabolism and the main source of ROS production. Meanwhile, dopaminergic neurons have high energy demands and are more vulnerable to mitochondrial dysfunction, which has been identified as a key candidate mechanism linking atmospheric aerosol exposure to PD‐relevant neurotoxicity [59]. Toxic components in atmospheric aerosols appear to directly target mitochondria in experimental models, suppress mitochondrial complex I activity, may trigger an energy metabolism crisis, and facilitate ROS generation, forming a self‐reinforcing vicious cycle [60]. Mitochondrial complex I (NADH‐coenzyme Q reductase) is a critical component of the mitochondrial respiratory chain, responsible for transferring electrons from NADH to coenzyme Q and participating in ATP synthesis. Metal particles (manganese, mercury) and polycyclic aromatic hydrocarbon metabolites in atmospheric aerosols may directly bind to subunits of mitochondrial complex I and suppress its activity, blocking the mitochondrial respiratory chain, interrupting electron transfer, and lowering ATP production. This may fail to satisfy the energy requirements of dopaminergic neurons and lead to neuronal dysfunction. Meanwhile, interrupted electron transfer leads to massive electron leakage, which combines with oxygen to generate ROS, further may harm mitochondrial structure and function—causing mitochondrial swelling, decreased membrane potential (ΔΨ), and mitochondrial DNA (mtDNA) damage. This ultimately may activate the mitochondrial apoptotic pathway and promote apoptosis of dopaminergic neurons in vitro and in vivo [61, 62]. Studies have confirmed that mitochondrial complex I activity is significantly decreased in the substantia nigra of PD patients, and similar mitochondrial dysfunction is observed in dopaminergic neurons of the substantia nigra in mice chronically exposed to atmospheric aerosols, indicating that inhibition of mitochondrial complex I is a critical step in PD may contributed by atmospheric aerosols [38].

#### 3.1.3 Interaction With PD‐Related Genes (e.g., PINK1, Parkin)

Oxidative stress and mitochondrial dysfunction may induced by atmospheric aerosols can also interact with PD‐related pathogenic genes (e.g., PINK1, Parkin), further amplifying neurotoxic effects and promoting the occurrence and development of PD. PINK1 and Parkin are important pathogenic genes associated with PD. The proteins they encode are mainly involved in mitophagy, which removes damaged mitochondria and maintains mitochondrial homeostasis. Mutations in the PINK1 or Parkin genes impair mitophagy, preventing the timely clearance of damaged mitochondria and leading to ROS accumulation and neuronal injury [63, 64].

Long‐term exposure to atmospheric aerosols elevates oxidative stress and causes mitochondrial damage, which in turn affects the PINK1/Parkin‐mediated mitophagy pathway: ROS can oxidatively modify PINK1 and Parkin proteins, inhibiting their activity and reducing mitophagy, resulting in the continuous accumulation of damaged mitochondria and further exacerbating oxidative stress and neuronal damage. Mitochondrial damage may induces the accumulation of PINK1 on the mitochondrial membrane, which recruits Parkin and initiates mitophagy. However, severe mitochondrial damage may induced by atmospheric aerosols exceeds the regulatory capacity of the PINK1/Parkin pathway, causing mitophagy dysfunction and ultimately leading to the apoptosis of dopaminergic neurons [65, 66]. In addition, individuals carrying susceptible loci of the PINK1 or Parkin genes have inherent defects in mitophagy. When exposed long‐term to atmospheric aerosols, they show lower tolerance to oxidative stress and mitochondrial damage, and their risk of PD is significantly increased [67], indicating that the interaction between environmental exposure and genetic factors plays an important role in the pathogenesis of PD.

### 3.2 Neuroinflammation and Abnormal Microglial Activation

#### 3.2.1 NLRP3 Inflammasome Activation and Release of IL‐1β, TNF‐α

Neuroinflammation is a key mechanism underlying dopaminergic neuron injury induced by atmospheric aerosols and also an important component of the pathological progression of PD, in which NOD‐like receptor family, pyrin domain containing 3 (NLRP3) inflammasome activation represents a central step in the neuroinflammatory response (Figure 3). The mechanistic evidence connecting aerosol toxicants to NLRP3 inflammasome activation originates from three separate research platforms with unequal translational reliability, which were previously integrated without explicit differentiation in the original manuscript. In vitro neuron‐microglia co‐culture models demonstrate that nano/ultrafine particles, transition metals and PAHs enter neural cells to trigger ROS overproduction and mitochondrial damage, which act as upstream priming signals to drive NLRP3 protein oligomerization, recruit ASC and pro‐caspase‐1, and assemble the functional NLRP3 inflammasome complex [68, 69]. Activated caspase‐1 cleaves pro‐IL‐1β and pro‐IL‐18 to produce mature pro‐inflammatory cytokines, and simultaneously initiates pyroptotic cell death to amplify local inflammatory responses in cell culture systems [70, 71]. These co‐culture assays isolate direct glial‐neuronal inflammatory crosstalk but cannot recapitulate systemic peripheral inflammation and lung‐brain barrier leakage observed in intact living organisms. Chronic intranasal and whole‐body inhalation mouse models further validate that aerosol exposure stimulates the NF‐κB signaling pathway in brain microglia, upregulating transcription of NLRP3, IL‐1β, and TNF‐α to sustain persistent central neuroinflammation [72].

Treated mice exhibit robust NLRP3 inflammasome activation within the substantia nigra, elevated pro‐inflammatory cytokine secretion and progressive dopaminergic neuron loss; transgenic mouse lines with glial‐specific NLRP3 knockout show markedly alleviated neuronal damage after aerosol exposure, confirming the pathogenic role of this inflammasome [73]. While animal models replicate the full physiological cascade of aerosol inhalation and brain inflammation, interspecies differences in microglial polarization and inflammatory signaling limit direct extrapolation to human central nervous system pathology. Human epidemiological cohort studies identify significantly higher circulating IL‐1β and TNF‐α levels in populations with long‐term PM2.5 exposure, and elevated peripheral inflammatory cytokine concentrations positively correlate with increased PD risk across multiple population data sets [74, 75]. However, human blood cytokine measurements only reflect systemic peripheral inflammation and cannot confirm direct NLRP3 inflammasome activation within human brain microglia, requiring complementary in vitro and animal toxicological data to establish causal inflammatory pathways. Toxic components in atmospheric aerosols (e.g., nano/ultrafine particles, metal particles, polycyclic aromatic hydrocarbons) can enter the brain, stimulate microglia and astrocytes, induce NLRP3 inflammasome activation, and subsequently elicit inflammatory responses with the release of large amounts of pro‐inflammatory cytokines, causing neuroinflammatory damage [76]. The NLRP3 inflammasome is a multiprotein complex composed of NLRP3 protein, apoptosis‐associated speck‐like protein containing a CARD (ASC), and caspase‐1, which can be activated by multiple signals including ROS, mitochondrial damage, and foreign particles [77]. ROS production and mitochondrial damage induced by atmospheric aerosols act as activating signals that induce NLRP3 protein oligomerization, recruit ASC and caspase‐1, and form the active NLRP3 inflammasome. Activated caspase‐1 cleaves pro‐IL‐1β and pro‐IL‐18 to generate mature pro‐inflammatory cytokines such as IL‐1β and IL‐18, and simultaneously induces pyroptosis, amplifying the inflammatory response [78]. Furthermore, atmospheric aerosol exposure can stimulate the nuclear factor‐κB (NF‐κB) signaling pathway, which promotes the transcription of inflammation‐related genes including NLRP3, IL‐1β, and TNF‐α, further enhancing the inflammatory response [79, 80]. These pro‐inflammatory cytokines directly damage dopaminergic neurons and recruit more inflammatory cell infiltration, establishing persistent neuroinflammation and accelerating PD‐related pathological progression.

#### 3.2.2 Microglial Polarization Toward the Pro‐Inflammatory Phenotype (M1)

Microglia are the resident immune cells in the brain and exhibit dual phenotypes: pro‐inflammatory (M1) and anti‐inflammatory (M2). Under normal physiological conditions, microglia are predominantly M2‐type, which clear foreign substances and apoptotic cells in the brain, secrete anti‐inflammatory factors, and maintain central nervous system homeostasis. Upon external stimulation such as atmospheric aerosols, microglia polarize toward the M1 phenotype and release large quantities of pro‐inflammatory factors, causing neuroinflammatory damage [81]. Toxic components in atmospheric aerosols may induce microglial M1 polarization by activating signaling pathways including TLR4/NF‐κB and JAK/STAT after entering the brain. Polarized M1 microglia secrete pro‐inflammatory factors and toxic mediators such as IL‐1β, TNF‐α, IL‐6, and inducible nitric oxide synthase (iNOS).

Among these, TNF‐α induces dopaminergic neuronal apoptosis, while iNOS catalyzes the production of large amounts of nitric oxide (NO), which reacts with ROS to form peroxynitrite (ONOO‐), further damaging dopaminergic neurons [82]. Meanwhile, M1 microglia can also damage dopaminergic neurons via phagocytosis, exacerbating neural injury. Studies have shown that mice chronically exposed to PM2.5 exhibit significantly may stimulated and M1‐polarized microglia in the substantia nigra, along with elevated pro‐inflammatory cytokine levels and reduced numbers of dopaminergic neurons. Microglial polarization toward the M1 phenotype is a critical step in atmospheric aerosol‐induced neuroinflammation and dopaminergic neuron damage [83].

#### 3.2.3 Astrocyte Response and Its Effects on Dopaminergic Neurons

Astrocytes are the most abundant glial cells in the brain, which are mainly responsible for maintaining the integrity of the BBB, providing nutritional support, and clearing neurotransmitters, thus playing an essential role in the survival and function of dopaminergic neurons. Atmospheric aerosol exposure can induce reactive activation of astrocytes, thereby affecting the survival of dopaminergic neurons. This effect is dual‐directional, but damage predominates under long‐term exposure [84]. After toxic components in atmospheric aerosols (e.g., nano/ultrafine particles, polycyclic aromatic hydrocarbons) enter the brain, they may induce astrocyte activation through signals such as ROS and inflammatory factors. Activated astrocytes can be classified into A1 phenotype (pro‐inflammatory) and A2 phenotype (anti‐inflammatory) [85]. Long‐term exposure to atmospheric aerosols drives astrocytes toward the A1 phenotype. A1 astrocytes secrete large amounts of pro‐inflammatory factors and toxic substances, inhibiting the survival of dopaminergic neurons. Meanwhile, they reduce neurotrophic support for dopaminergic neurons by decreasing the secretion of neurotrophic factors such as brain‐derived neurotrophic factor (BDNF). In addition, may stimulated astrocytes produce abundant glial fibrillary acidic protein (GFAP) and form glial scars, which impede axonal regeneration and synaptic connectivity of dopaminergic neurons, further exacerbating neural injury [86, 87]. Astrocyte activation also impairs BBB integrity and increases barrier permeability, allowing more toxic components of atmospheric aerosols to enter the brain, forming a vicious cycle.

### 3.3 Protein Homeostasis Imbalance and Abnormal Aggregation

#### 3.3.1 Conformational Alteration, Oligomerization, and Fibrosis of α‐syn

Protein homeostasis imbalance is one of the core pathological features of PD. Among them, conformational alteration, oligomerization, and fibrosis of α‐syn with the formation of Lewy bodies represent the most typical pathological hallmark of PD. Atmospheric aerosol exposure can significantly disturb protein homeostasis and may induce abnormal aggregation of α‐syn [88] (Figure 3). α‐syn is a soluble small protein that, under normal physiological conditions, is mainly distributed in the presynaptic membrane and participates in dopamine release and synaptic function regulation. Upon external stimulation, α‐syn undergoes conformational changes from a soluble α‐helical structure to an insoluble β‐sheet structure, followed by oligomerization to form oligomers, and eventually aggregates into fibrillar bundles known as Lewy bodies [89]. Various toxic components in atmospheric aerosols may induce abnormal aggregation of α‐syn: Nano/ultrafine particles can directly bind to α‐syn and may induce its conformational change and oligomerization. For example, PM2.5 may elicit conformational changes in α‐syn fibrils to form more toxic specific variants with markedly enhanced stability, aggregation ability, and neurotoxicity, and this effect is global. Metal particles (copper, iron) can bind to the binding sites of α‐syn and promote its oligomerization and fibrosis. Organic pollutants such as polycyclic aromatic hydrocarbons can damage the structure of α‐syn via oxidative stress and may induce its abnormal aggregation [90, 91, 92]. Furthermore, oxidative stress and mitochondrial dysfunction may induced by atmospheric aerosols further aggravate the abnormal aggregation of α‐syn. Abnormally aggregated α‐syn, in turn, disturbs protein homeostasis by inhibiting the proteasome and autophagy‐lysosomal system. Meanwhile, its oligomeric and fibrillar products exert strong neurotoxicity, directly damaging dopaminergic neurons and promoting the occurrence and development of PD [93]. Studies have confirmed that humans and animal models chronically exposed to atmospheric aerosols exhibit significantly increased abnormal α‐syn aggregation and more Lewy bodies in the substantia nigra, which are positively correlated with the degree of dopaminergic neuron injury [94, 95].

#### 3.3.2 Impaired Function of the Proteasome and Autophagy‐Lysosomal System

The proteasome system and autophagy‐lysosomal system are the two core systems in the body responsible for clearing abnormally aggregated proteins and maintaining protein homeostasis. When the functions of these two systems are impaired, abnormally aggregated proteins cannot be cleared in a timely manner, leading to their accumulation in cells and subsequent cellular damage. Atmospheric aerosol exposure can significantly damage the functions of the proteasome and autophagy‐lysosomal system, resulting in protein homeostasis imbalance, which in turn promotes abnormal α‐syn aggregation and dopaminergic neuron injury.

The proteasome system is mainly responsible for clearing short‐lived, oxidatively modified, or abnormally folded proteins. ROS production may induced by atmospheric aerosols can oxidatively modify the subunits of the proteasome, inhibiting its activity and preventing it from effectively clearing abnormally folded α‐syn, thereby promoting α‐syn oligomerization and aggregation. Meanwhile, inflammatory factors (such as TNF‐α and IL‐1β) can also inhibit proteasome activity, further exacerbating protein homeostasis imbalance [96, 97].

The autophagy‐lysosomal system is mainly responsible for clearing long‐lived proteins, abnormally aggregated proteins, and damaged organelles. Toxic components in atmospheric aerosols (such as metal particles, nano/ultrafine particles, and chlorpyrifos) can damage the autophagy‐lysosomal system through multiple pathways: inhibiting autophagy initiation signals [such as the Adenosine 5’‐monophosphate (AMP)‐activated protein kinase (AMPK)/mammalian target of rapamycin (mTOR) signaling pathway] to reduce autophagosome formation; damaging the integrity of the lysosomal membrane, leading to the release of lysosomal enzymes into the cytoplasm, which impairs cellular structure and function, and simultaneously inhibiting the fusion of autophagosomes with lysosomes, resulting in blocked autophagic flux; inducing a decrease in lysosomal enzyme activity, preventing effective degradation of abnormal proteins and damaged organelles within autophagosomes [98, 99, 100]. For example, chlorpyrifos exposure can significantly inhibit autophagic flux, leading to abnormal accumulation of α‐syn and subsequent loss of dopaminergic neurons, while enhancing autophagic flux can effectively protect neurons from chlorpyrifos‐induced damage [101].

#### 3.3.3 Potential Promotion of Intercellular Transmission

Atmospheric aerosol exposure not only induces abnormal intracellular α‐syn aggregation but also promotes the intercellular transmission of abnormally aggregated α‐syn, leading to the spread of neural injury, which is an important reason for the progressive pathological progression of PD. Abnormally aggregated α‐syn (especially oligomers) can be released from damaged dopaminergic neurons into the extracellular microenvironment through various ways, and then be taken up by surrounding healthy dopaminergic neurons, microglia, and astrocytes. This further induces conformational changes and abnormal aggregation of endogenous α‐syn in these cells, forming a “domino effect” and causing widespread spread of neural injury. Atmospheric aerosol exposure can promote the intercellular transmission of α‐syn through multiple mechanisms: inducing cell apoptosis or necrosis, leading to cell rupture and release of large amounts of abnormal α‐syn; activating intracellular secretory pathways to promote the release of abnormal α‐syn into the extracellular space through exocytosis; damaging synaptic connections between cells, increasing the permeability of the extracellular space, and providing favorable conditions for the diffusion of abnormal α‐syn; inducing microglial activation, and after phagocytosing abnormal α‐syn, microglia may release it into the extracellular space, further promoting its transmission [102, 103, 104, 105]. In addition, neuroinflammation and BBB damage induced by atmospheric aerosols can also increase the disorder of the brain microenvironment, promoting the transmission of abnormal α‐syn between different brain regions, leading to the gradual spread of PD pathological changes from the olfactory bulb and substantia nigra to the cerebral cortex and other regions, may eliciting more extensive neurological dysfunction.

### 3.4 Alterations in Epigenetic Regulation

#### 3.4.1 Regulation of Neurotoxicity‐Related Genes by DNA Methylation

Epigenetic regulation refers to the modulation of gene transcription and expression without altering the DNA sequence, via mechanisms including DNA methylation, histone modification, and noncoding RNA regulation, thereby governing cellular physiological and pathological processes (Figure 3). Exposure to atmospheric aerosols can alter DNA methylation levels, regulate the expression of neurotoxicity‐related genes, and further induce dopaminergic neuron damage, contributing to the initiation and progression of PD [83]. DNA methylation occurs mainly at CpG islands in gene promoter regions and generally leads to transcriptional silencing; its aberrant changes are closely associated with various neurodegenerative diseases. Toxic components in atmospheric aerosols (e.g., polycyclic aromatic hydrocarbons, metal particles) can modify the methylation status of neurotoxicity‐related genes by affecting the activities of DNA methyltransferases (DNMTs) and TET demethylases. On one hand, they can elevate DNMT activity, increasing methylation at CpG islands in the promoter regions of PD‐related protective genes (e.g., BDNF, PINK1, Parkin), suppressing their transcription and expression, and weakening their neuroprotective effects on dopaminergic neurons. On the other hand, they can inhibit TET demethylase activity, reducing methylation of pro‐inflammatory and pro‐apoptotic genes (e.g., IL‐1β, TNF‐α, Bax), promoting their expression, and exacerbating neuroinflammation and neuronal apoptosis [106, 107, 108]. For instance, exposure to polycyclic aromatic hydrocarbons increases methylation in the BDNF promoter region, represses BDNF expression, reduces trophic support for dopaminergic neurons, and causes neuronal injury. Exposure to manganese alters the methylation status of the Parkin gene, inhibits its expression, impairs mitophagy, and aggravates mitochondrial damage and oxidative stress [109, 110].

#### 3.4.2 Changes in Noncoding RNA (e.g., miRNA) Expression Profiles

Noncoding RNAs (ncRNAs) are a class of RNA molecules that do not encode proteins. Among them, microRNAs (miRNAs) are the most extensively studied, which regulate gene expression by binding to the 3’‐untranslated regions of target mRNAs, inhibiting translation or promoting mRNA degradation. MiRNAs play critical roles in the development, survival, and functional maintenance of dopaminergic neurons. Exposure to atmospheric aerosols can significantly alter miRNA expression profiles in the brain, induce neurotoxicity via target gene regulation, and promote PD pathogenesis [111]. Numerous studies have shown that humans and animal models chronically exposed to atmospheric aerosols (e.g., PM2.5) exhibit markedly abnormal expression of multiple miRNAs in the substantia nigra, some of which are closely linked to PD pathogenesis [112, 113]. For example, miR‐7 and miR‐133b are significantly downregulated in the brains of PD patients. MiR‐7 targets and inhibits α‐synuclein expression to reduce its abnormal aggregation, while miR‐133b regulates the differentiation and survival of dopaminergic neurons. Aerosol exposure downregulates both miRNAs, releasing the inhibition of α‐synuclein and impairing dopaminergic neuron survival, thus exacerbating neural injury [114, 115]. In addition, inflammation‐associated miRNAs such as miR‐146a and miR‐155 are significantly upregulated following aerosol exposure; they enhance neuroinflammation by promoting pro‐inflammatory cytokine expression, further damaging dopaminergic neurons [116, 117]. These findings suggest that aberrant miRNA expression profiles may represent a key epigenetic mechanism underlying atmospheric aerosol‐induced PD, and hold promise as potential targets for early diagnosis and therapy of PD.

#### 3.4.3 Roles of Histone Modifications in Sustained Inflammatory Responses

Histone modification is another major epigenetic regulatory mechanism, mainly including acetylation, methylation, and phosphorylation, which affect gene transcription and expression by remodeling chromatin structure. Histone acetylation is generally associated with transcriptional activation, whereas histone methylation exerts diverse effects, either promoting or inhibiting transcription. Its dysregulation is closely related to neuroinflammation and neuronal damage. Exposure to atmospheric aerosols can modulate the expression of inflammation‐related genes by altering histone modification patterns, sustaining chronic neuroinflammation, and thereby inducing dopaminergic neuron injury [83, 118]. Toxic components in atmospheric aerosols (e.g., nano/ultrafine particles, polycyclic aromatic hydrocarbons) can modify histone modification levels by activating relevant modifying enzymes. On one hand, they activate histone deacetylases (HDACs), reducing histone acetylation, suppressing the transcription of anti‐inflammatory genes and neurotrophic factor genes, and promoting the transcription of pro‐inflammatory genes to amplify neuroinflammation. On the other hand, they may stimulate histone methyltransferases, inducing abnormal levels of histone modifications such as H3K4me3 and H3K27me3, regulating the expression of inflammation‐related genes (e.g., NLRP3, IL‐1β) and maintaining persistent neuroinflammation [119, 120, 121]. For example, PM2.5 exposure may stimulates HDACs, decreases histone acetylation, inhibits BDNF expression, and upregulates pro‐inflammatory genes including IL‐1β and TNF‐α, worsening neuroinflammation and dopaminergic neuron damage. Furthermore, histone modifications can interact with DNA methylation and miRNA regulation to form a complex epigenetic regulatory network, collectively mediating atmospheric aerosol‐induced neurotoxicity [122, 123]. Studies have revealed that soot nanoparticles can promote ferroptosis in dopaminergic neurons by regulating m6A RNA methylation, further enriching the epigenetic mechanisms of atmospheric aerosol‐induced PD [124].

### 3.5 Crosstalk and Cascade Amplification of Key Molecular Pathways

#### 3.5.1 Oxidative Stress‐Neuroinflammation Positive Feedback Loop

**Figure 4:**
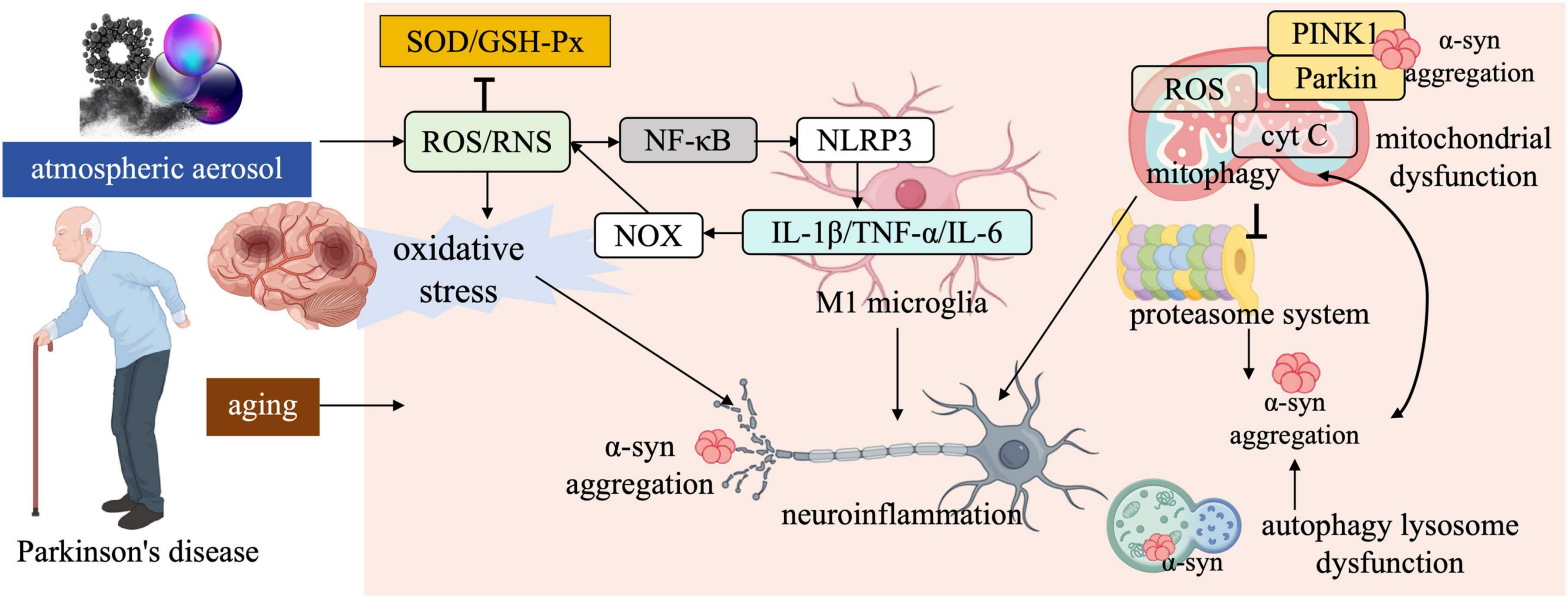
Mechanistic pathways linking atmospheric aerosol exposure to PD pathogenesis. Atmospheric aerosol exposure acts synergistically with aging to promote PD pathogenesis via interconnected molecular and cellular mechanisms. Aerosols induce the generation of ROS/RNS, which can overwhelm antioxidant defenses such as SOD and GSH‐Px. Excess ROS/RNS directly promotes α‑syn aggregation and may stimulates the NF‑κB signaling pathway. Activated NF‑κB further upregulates the NLRP3 inflammasome in M1 microglia, may eliciting the release of pro‑inflammatory cytokines including IL‑1β, TNF‑α, and IL‑6. These cytokines stimulate NOX, forming a positive feedback loop that amplifies oxidative stress and neuroinflammation, further worsening α‑syn aggregation and neuronal damage. Meanwhile, accumulated ROS/RNS impair mitochondrial function, inhibit PINK1/Parkin‑mediated mitophagy and promote cyt C release, while also compromising the proteasome and autophagy‑lysosome pathways, reducing the clearance of misfolded α‑syn. Together, these cascading pathways converge to drive dopaminergic neuron degeneration and the progression of PD.

Oxidative stress and neuroinflammation do not act independently; they interact to form a positive feedback loop, which collectively amplifies the neurotoxic effects induced by atmospheric aerosols. This is a key driver for the continuous progression of PD pathogenesis (Figure 4). After toxic components in atmospheric aerosols enter the brain, they first may elicit a burst of ROS/RNS production and induce oxidative stress. Oxidative stress not only directly damages dopaminergic neurons but also acts as an activation signal to initiate neuroinflammation [125]. ROS can may stimulate the NF‐κB signaling pathway, promote the activation of the NLRP3 inflammasome, induce microglial polarization toward the M1 phenotype, and release large amounts of pro‐inflammatory cytokines such as IL‐1β and TNF‐α, thereby may eliciting neuroinflammation. These pro‐inflammatory factors can further promote ROS production by activating enzymes such as NOX and inhibit the antioxidant defense system, aggravating oxidative stress. In this way, a self‐reinforcing positive feedback loop is formed, in which oxidative stress and neuroinflammation mutually exacerbate and amplify each other [126, 127]. For example, PM2.5 exposure induces ROS generation, may stimulates the NLRP3 inflammasome, and releases IL‐1β. IL‐1β in turn upregulates NOX expression, increases ROS production, and inhibits the activities of SOD and GSH‐Px, further worsening oxidative stress and thereby exacerbating dopaminergic neuron damage and α‐syn aggregation [128]. The formation of this positive feedback loop continuously amplifies aerosol‐induced neural injury and accelerates the pathological progression of PD.

#### 3.5.2 Vicious Cycle Between Mitophagy Deficiency and Protein Aggregation

A close interaction exists between mitophagy deficiency and abnormal aggregation of α‐syn forming a vicious cycle that further aggravates atmospheric aerosol‐induced dopaminergic neuron damage. Atmospheric aerosol exposure can simultaneously may elicit mitophagy deficiency and α‐syn aggregation, and these two pathological processes promote each other to form a vicious cycle [129]. On the one hand, mitophagy deficiency prevents the timely removal of damaged mitochondria, which produce excessive ROS and release pro‐apoptotic factors such as cytochrome c (cyt C). ROS may drive conformational shifts, oligomerization, and fibrillization of α‐syn, promoting its abnormal aggregation, while also inhibiting the proteasome and autophagy‐lysosome system, further worsening protein aggregation. On the other hand, abnormally aggregated α‐syn inhibits mitophagy through multiple pathways: α‐syn binds to PINK1 and Parkin and suppresses their activities, blocking the initiation of mitophagy. Meanwhile, aggregated α‐syn deposits on the mitochondrial membrane, impairing mitochondrial structure and function and further exacerbating mitophagy deficiency [130, 131]. For instance, chlorpyrifos exposure inhibits autophagic flux, leading to abnormal α‐syn accumulation, which in turn further impairs the autophagy–lysosome system and damages mitochondria, causing mitophagy deficiency and increased ROS production, thereby promoting more α‐syn aggregation. Metal particle‐induced mitochondrial damage inhibits PINK1/Parkin‐mediated mitophagy and increases ROS, promoting α‐syn aggregation, which further damages mitochondria, completing the vicious cycle [101, 132]. This vicious cycle progressively worsens dopaminergic neuron injury, eventually leading to neuronal degeneration and death and the onset of PD‐related pathological changes.

### 3.6 Multiple‐Hit Hypothesis: Synergistic Effects of Aerosol Components and Genetic Susceptibility

The development of PD is not caused by a single factor but by the combined action of multiple factors. Specifically, the synergistic effects of atmospheric aerosol components and superimposed genetic susceptibility are consistent with the “multiple‐hit hypothesis” of PD pathogenesis. This hypothesis holds that individuals with genetic susceptibility undergo cumulative damage from multiple environmental “hits,” eventually leading to PD. As a complex environmental exposure, atmospheric aerosols exert their multiple toxic components synergistically, and together with individual genetic susceptibility, constitute the “multiple hits” in PD development [133]. First, synergistic toxic effects occur among various toxic components in atmospheric aerosols (metal particles, POPs, nano/ultrafine particles), and their combined toxicity is far greater than that of individual components [107]. For example, nano/ultrafine particles can adsorb metal particles and polycyclic aromatic hydrocarbons, enhancing their transport and accumulation in vivo. Meanwhile, oxidative stress induced by metal particles can enhance the metabolic activation of polycyclic aromatic hydrocarbons and amplify their neurotoxicity [134]. The synergistic action of metal particles and POPs further exacerbates mitochondrial dysfunction, α‐syn aggregation, and neuroinflammation, jointly promoting dopaminergic neuron damage [135]. Second, atmospheric aerosol exposure and individual genetic susceptibility have a superimposed effect. People carrying PD‐related pathogenic genes (e.g., PINK1, Parkin, SNCA) or susceptibility loci have inherent defects in dopaminergic neurons and lower tolerance to aerosol‐induced oxidative stress, mitochondrial damage, and neuroinflammation. Upon long‐term aerosol exposure, their risk of PD increases significantly, with earlier onset and faster progression [136]. In addition, aging is an important component of the “multiple hits.” With advancing age, the antioxidant system, mitochondrial function, and autophagy–lysosome system decline, while immune function becomes dysregulated, increasing sensitivity to aerosol toxicity. The combination of aerosol exposure and aging further accelerates PD development [11]. For example, young and middle‐aged adults with long‐term exposure to environmental risk factors such as atmospheric aerosols show a significantly elevated PD risk, with different progression patterns compared to elderly‐onset patients, further supporting the superimposed effects of environmental exposure and aging [11, 53].

## 4 Research Methods and Models

### 4.1 In Vitro Models: Cell Lines, Primary Neurons, and Neuron‐Glia Co‐Culture Exposure Models

In vitro models represent fundamental tools for investigating the molecular mechanisms of neurotoxicity related to PD induced by atmospheric aerosols. They offer advantages including simple operation, low cost, and precise control over exposure conditions. The main in vitro models comprise cell line models, primary neuron models, and glial cell co‐culture models. These systems can may recapitulate partial direct interactions between aerosol toxic components and neural cells, facilitate mechanistic investigations, and support the screening of potential intervention agents, providing a basis for subsequent in vivo studies. In typical in vitro experiments, atmospheric aerosol samples are prepared and administered to cell lines at various concentrations and durations. Neurotoxicity and underlying molecular mechanisms are then evaluated by measuring indicators such as cell viability, apoptosis rate, ROS levels, mitochondrial membrane potential, inflammatory cytokine expression, and α‐syn aggregation [137]. Primary neuron models, such as cultures derived from the midbrain substantia nigra or cerebral cortex of rodents, particularly primary dopaminergic neurons, exhibit physiological functions closer to in vivo neurons and can more realistically recapitulate aerosol‐induced neuronal injury [122, 138]. However, these models are limited by complicated isolation procedures, low cell survival rates, and relatively high costs. Glial cell co‐culture models (e.g., co‐culture of dopaminergic neurons with microglia or astrocytes) mimic neuron‐glia crosstalk within the central nervous system in vivo, enabling investigations into the synergistic mechanisms underlying aerosol‐induced neuroinflammation and neuronal damage [139]. For instance, neuron‐microglia co‐cultures allow the study of indirect damage to dopaminergic neurons following aerosol‐induced microglial activation [140]. This model is also applicable to exploring intercellular signaling between neurons and glia, as well as the cell‐to‐cell propagation of α‐syn.

### 4.2 In Vivo Models: Inhalation Exposure and Intranasal Instillation Models in Rodents

In vivo models are essential tools for investigating the neurotoxicity related to PD induced by atmospheric aerosols. They can simulate the transport, accumulation, and toxic effects of aerosols in the body, and thus better reflect real human exposure scenarios. Inhalation exposure models most closely mimic the actual exposure route in humans. By establishing animal whole‐body or nose‐only inhalation systems, animals are exposed to atmospheric aerosol samples or standard particulate matter (e.g., PM2.5, nanoparticles) at defined concentrations and durations to simulate long‐term human inhalation [141, 142, 143]. This model can authentically reflect the distribution, transport, and accumulation of toxic aerosol components in vivo, as well as their damage to the respiratory and central nervous systems. Pathological and molecular mechanisms can be elucidated by evaluating behavioral performance, the number of dopaminergic neurons in the substantia nigra, α‐syn aggregation, inflammatory cytokine expression, and other indicators. For example, mice exposed to aerosolized chlorpyrifos exhibit impaired motor coordination, loss of dopaminergic neurons in the substantia nigra pars compacta, accompanied by abnormal α‐syn aggregation and microglial activation, can partially recapitulate PD‐like pathological alterations under controlled exposure conditions [144]. However, this model is limited by complex equipment, high cost, and difficulties in precisely controlling exposure concentration and duration. Intranasal instillation models are simple and efficient. By instilling a fixed dose of aerosol extract or particle suspension into the nasal cavity of animals, the direct brain entry of aerosols via the olfactory pathway can be simulated, making it suitable for studying neurotoxic mechanisms through this route [145]. This model features easy operation, low cost, and precise control over dose and exposure time. It is mainly used to explore pathological injury in the olfactory pathway, early changes in α‐syn aggregation, and the distribution and transport of toxic components in the brain [90]. In addition, the combination of PD transgenic animal models (e.g., α‐syn transgenic mice, PINK1/Parkin knockout mice) with the above exposure models allows investigation of the interaction between atmospheric aerosol exposure and genetic factors. It can clarify differences in susceptibility to aerosol neurotoxicity among animals with different genetic backgrounds, further illuminating the pathogenesis of PD [83, 90].

### 4.3 Human Studies: Biomarkers, Imaging, and Epidemiological Association Analysis

Human studies are critical for confirming the association between atmospheric aerosol exposure and PD. Mainly based on epidemiological analysis, biomarker detection, and neuroimaging, these studies explore the relationships between aerosol exposure and PD risk or disease progression, validate molecular mechanisms identified in vitro and in vivo, and provide a scientific basis for risk assessment and early warning of PD [146, 147]. Epidemiological association analyses mainly include cohort studies, case‐control studies, and cross‐sectional studies. By collecting individual aerosol exposure history (e.g., long‐term ambient air quality at residence, occupational exposure), combined with PD incidence, the association between aerosol exposure and PD risk, as well as dose–response relationships related to exposure level and duration, can be analyzed [8, 148]. For instance, a case‐control study in agricultural areas of California, United States, showed that long‐term occupational exposure to chlorpyrifos significantly increased PD risk, with exposure 10–20 years before diagnosis showing a stronger association. A prospective study of 346 PD patients demonstrated that long‐term exposure to high concentrations of PM2.5 and NO_2_ was associated with elevated PD risk [23, 74]. Furthermore, epidemiological studies can evaluate the links between aerosol exposure and clinical symptoms or disease progression, supporting evidence for disease prevention. Biomarker detection measures indicators in blood, cerebrospinal fluid, urine, nasal secretions, and other biofluids as markers linking aerosol exposure and PD‐related pathological changes. Exposure biomarkers mainly include toxic components of atmospheric aerosols (e.g., metal particles, PAH metabolites) to assess population exposure levels. Effect biomarkers include oxidative stress indices [malondialdehyde (MDA), SOD], inflammatory cytokines (IL‐1β, TNF‐α), α‐syn, miRNAs, and others, reflecting exposure‐induced tissue damage and PD progression [149, 150]. For example, decreased homovanillic acid (HVA) in cerebrospinal fluid can serve as an auxiliary diagnostic marker for PD, while elevated blood inflammatory cytokines can reflect neuroinflammation induced by atmospheric aerosols [151, 152]. Neuroimaging techniques (brain magnetic resonance imaging [MRI], positron emission tomography‐computed tomography [PET‐CT], etc.) enable evaluation of dopaminergic neuron density in the substantia nigra, α‐syn aggregation, cerebral atrophy, and other structural or functional changes, allowing assessment of brain injury caused by aerosol exposure and providing imaging support for early PD diagnosis [153, 154].

### 4.4 Emerging Technologies: Applications of Multiomics Integration Analysis, Brain Organoids, and Single‐Cell Sequencing

With the rapid development of life science technologies, emerging tools including multiomics integration analysis, brain organoids, and single‐cell sequencing have been increasingly applied to studies of neurotoxicity related to PD induced by atmospheric aerosols. These approaches provide novel methods and insights for elucidating the core molecular mechanisms, effectively compensate for the limitations of conventional research strategies, and significantly advance the environmental etiology of PD.

Multiomics integration analysis covers genomics, transcriptomics, proteomics, metabolomics, and epigenomics. Through combined multiomics detection of cells, animal models, or human samples after exposure, this strategy systematically screens differentially expressed genes, proteins, metabolites, and epigenetic modification sites, and constructs multidimensional molecular regulatory networks, thereby revealing the complex molecular mechanisms underlying PD induced by atmospheric aerosols [155, 156]. This technique can identify key signaling pathways (e.g., oxidative stress, neuroinflammation, mitophagy) and epigenetic regulatory patterns, and explore potential intervention targets and early biomarkers at a holistic level.

Brain organoids are three‐dimensional neural tissue models differentiated from pluripotent stem cells, which highly recapitulate the morphological structure, physiological function, neural development, and pathological alterations of the human brain. They provide a research system closer to the real physiological state of the human brain for investigating aerosol‐induced neurotoxicity [157, 158]. Compared with traditional in vitro cell models and animal models, brain organoids can more realistically reproduce the transport, accumulation, and biological effects of toxic components in the human brain, restore the interaction networks among dopaminergic neurons, microglia, and astrocytes, as well as the abnormal aggregation and intercellular propagation of α‐syn, offering an innovative platform for mechanistic dissection and drug screening.

Single‐cell sequencing enables precise characterization of omic profiles at the single‐cell level, reveals high heterogeneity within tissues and cell populations, and identifies distinct molecular responses among different cell types or even subpopulations following aerosol exposure. This technology can identify signature genes of polarized microglial subsets, specific damage mechanisms in dopaminergic subpopulations, and explore the intercellular transmission of α‐syn, as well as cell‐specific interactions between aerosol exposure and genetic susceptibility. It thus provides a scientific basis for precise subtyping, early warning, and targeted intervention of PD. Various in vitro, in vivo and human population research systems have been established to explore aerosol‐related neurotoxicity. The typical exposure schemes, key detection indicators, merits and translational bottlenecks of mainstream experimental models are consolidated in Table 2 for intuitive comparison.

**Table 2:** Experimental models for aerosol‐associated PD neurotoxicity: Exposure, outcomes, strengths, and limitations.

Model category	Specific experimental system	Typical aerosol exposure setup	Core detected pathological outcomes	Main strengths	Critical limitations
In vitro monolayer cell models [137, 138]	SH‐SY5Y dopaminergic cell line, BV2 microglia, primary astrocytes	Aerosol extracts/PM suspensions at gradient concentrations, short‐term incubation (6–72 h)	ROS elevation, mitochondrial membrane potential loss, α‐syn aggregation, pro‐inflammatory cytokine secretion	Low cost, high throughput, precise concentration control, single cell‐type mechanism dissection	Lack cell‐cell crosstalk; cannot simulate systemic circulation, olfactory/respiratory entry pathways; short‐term exposure fails to mimic lifelong low‐dose damage
In vitro co‐culture models [139, 140]	Dopaminergic neuron + microglia/astrocyte triple co‐culture	Same particulate exposure as monolayer; long‐term subchronic treatment	M1/A1 glial polarization, indirect neuronal inflammatory injury, intercellular α‐syn transmission	Recapitulates neuron‐glia interaction; suitable for neuroinflammation research	Still absent in vivo respiratory‐brain transport, immune circulation feedback
3D brain organoids [157, 158]	Human pluripotent stem cell‐derived midbrain organoids with dopaminergic neurons	Static particulate immersion or air‐liquid interface exposure	α‐syn fibril formation, mitochondrial dysfunction, epigenetic modification shifts	Humanized neural tissue structure; recapitulate human‐specific α‐syn pathology; no animal ethical issues	High culture cost; no intact BBB and systemic organ circulation; hard to simulate inhalation intake
In vivo intranasal instillation rodent model [83, 90]	Wild‐type/PINK1/Parkin/α‐syn transgenic mice	Fixed‐volume aerosol suspension dripped into nasal cavity, repeated chronic administration	Olfactory bulb particle deposition, early hyposmia, substantia nigra neuronal loss, microglial activation	Low equipment cost; specifically simulate olfactory brain‐entry pathway; dose accurately controllable	Deviates from natural respiratory inhalation; bypasses alveolar absorption and systemic inflammatory cascade
In vivo whole‐body/nose‐only inhalation model [141, 142, 143]	C57BL/6, PD gene knockout/transgenic mice	Continuous chamber exposure to real ambient aerosols or standard PM2.5/nanoparticles (weeks–months)	Systemic inflammatory cytokines, BBB leakage, progressive dopaminergic degeneration, motor dysfunction	Closely mimic real human inhalation exposure; reproduce lung‐brain systemic inflammation cascade	Expensive inhalation equipment; difficult to stabilize real‐world aerosol component composition; high animal consumption
Human population research [8, 148]	Cohort/case‐control/cross‐sectional cohorts	Retrospective residential/occupational long‐term aerosol exposure assessment	PD incidence risk ratio, blood/CSF biomarkers (α‐syn, inflammatory factors), brain MRI/PET imaging	Highest translational value; reflect real human multifactor mixed exposure	Multiple confounding variables (genetics, lifestyle, other pollutants); individual exposure dose inaccurate; extremely long follow‐up cycles
Multiomics + single‐cell platform [155, 156]	Human/animal tissue, primary cells postparticulate exposure	Same exposure schemes as above	Transcriptome/proteome/epigenome differential profiles, single‐cell glia/neuron subtype toxic signatures	Unbiased high‐throughput pathway screening; identify cell‐type‐specific damage mechanisms	Omics data interpretation complex; most discoveries lack subsequent functional verification

### 4.5 Potential Intervention Targets and Strategic Prospects

#### 4.5.1 Targeting Core Mechanisms: Antioxidants, Anti‐Inflammatory Agents, and Autophagy Enhancers

Based on the core molecular mechanisms underlying PD induced by atmospheric aerosols (oxidative stress, neuroinflammation, disrupted protein homeostasis, and mitochondrial dysfunction), targeted intervention targets can be identified and therapeutic agents developed, providing novel strategies for the treatment of aerosol‐related PD. Current potential interventions mainly include antioxidants, anti‐inflammatory drugs, and autophagy enhancers, which can alleviate aerosol‐induced neurotoxicity and protect dopaminergic neurons by regulating core molecular pathways. Antioxidants represent important neuroprotective agents that attenuate oxidative stress injury by scavenging excessive ROS and RNS and restoring redox balance. They are classified into natural and synthetic categories. Natural antioxidants (curcumin, resveratrol, vitamins C and E) exhibit low toxicity and high safety, protecting dopaminergic neurons through multiple pathways. Among synthetic antioxidants, edaravone has been clinically used for PD treatment, effectively scavenging ROS and reducing neuronal damage. In addition, metal chelators (deferoxamine, penicillamine) can sequester excess metal ions, reduce ROS generation, and mitigate neurotoxicity induced by metal particles [159, 160, 161].

Anti‐inflammatory agents protect neurons by suppressing neuroinflammation, reducing abnormal glial activation, and inhibiting pro‐inflammatory cytokine release. Intervention targets mainly focus on the NLRP3 inflammasome, NF‐κB signaling, and microglial polarization. MCC950 specifically inhibits NLRP3 inflammasome activation, while pyrrolidine dithiocarbamate (PDTC) suppresses NF‐κB signaling and modulates glial polarization, both of which alleviate neural injury. Natural anti‐inflammatory compounds such as triptolide and apigenin show high safety and mild side effects, exerting neuroprotection by regulating inflammatory pathways and showing promise for preventive and adjuvant therapy [162, 163, 164].

Autophagy enhancers protect neurons by restoring the autophagy‐lysosomal system, clearing abnormally aggregated proteins, and improving defective mitophagy. Rapamycin inhibits the mTOR pathway to initiate autophagy, accelerating α‐synuclein degradation and enhancing mitophagy. Chloroquine derivatives such as hydroxychloroquine modulate lysosomal function and strengthen autophagic clearance [165, 166]. Furthermore, curcumin and resveratrol possess combined antioxidant, anti‐inflammatory, and autophagy‐promoting activities, enabling multitarget synergistic neuroprotection and expanding therapeutic options.

#### 4.5.2 Targeting Epigenetic Regulation: DNA Methylation Inhibitors, miRNA Mimics/Inhibitors

Targeting epigenetic regulation has emerged as a new frontier in PD therapy, based on epigenetic alterations induced by atmospheric aerosols. Major strategies include DNA methylation inhibitors, miRNA mimics/inhibitors, and histone modification modulators, which alleviate neurotoxicity by reversing aberrant epigenetic modifications and restoring normal expression of neurotoxicity‐related genes. DNA methylation inhibitors exert neuroprotection by inhibiting DNA methyltransferase (DNMT) activity, reducing promoter methylation of PD‐related protective genes such as BDNF and PINK1 to restore their expression, while increasing methylation of pro‐inflammatory and pro‐apoptotic genes to suppress their abnormal expression [167, 168]. For example, 5‐azacytidine (5‐Aza) and 5‐aza‐2'‐deoxycytidine (5‐Aza‐CdR) markedly inhibit DNMT activity, promote BDNF expression, and reduce pro‐inflammatory cytokine release, demonstrating neuroprotective effects in both in vitro and in vivo models [169]. However, these agents suffer from low specificity and potential genomic instability with long‐term use, requiring the development of more selective compounds to improve precision and safety.

miRNA‐targeted interventions correct dysregulated miRNA expression caused by aerosol exposure by delivering mimics or inhibitors, thereby regulating target gene expression and reducing neurotoxicity [170]. For downregulated miR‐7 and miR‐133b, mimics restore expression to reduce α‐syn aggregation and promote dopaminergic neuronal survival. For upregulated miR‐146a and miR‐155, inhibitors suppress expression and alleviate neuroinflammation. This strategy is currently in preclinical development for PD, with high specificity as a key advantage, although brain delivery efficiency and off‐target effects remain to be addressed. Histone modification modulators regulate the expression of inflammation‐related genes and neurotrophic factors by adjusting histone acetylation and methylation, thereby reducing neural damage. Histone deacetylase (HDAC) inhibitors such as valproic acid (VPA) and trichostatin A (TSA) increase histone acetylation, enhance neurotrophic factor expression, and suppress pro‐inflammatory genes. Histone methyltransferase inhibitors correct aberrant histone methylation and relieve chronic neuroinflammation [171, 172, 173]. Combined use of these modulators with DNA methylation inhibitors and miRNA‐targeted agents can form a more effective epigenetic intervention network, enhancing overall neuroprotective efficacy.

#### 4.5.3 Public Health Interventions: Source Control and Population Protection against Aerosol Exposure

In addition to pharmacological interventions, public health measures targeting atmospheric aerosol exposure—reducing aerosol emissions through source control and lowering exposure levels via population protection—represent the most promising primary preventive strategies hypothesized to reduce PD burden linked to aerosol exposure, and also represent an important research direction in the field of public health. The core of source control for aerosol exposure is to reduce anthropogenic emissions. Priority should be given to controlling major emission sources such as fossil fuel combustion, industrial production, and agricultural activities. This includes strengthening industrial waste gas treatment, promoting clean energy (e.g., solar energy, wind energy) to replace fossil fuels, reducing vehicle exhaust through the popularization of new energy vehicles and optimized traffic management, regulating agricultural activities (banning crop straw burning and rational use of pesticides), and enhancing indoor pollution control to reduce indoor sources such as cooking fumes and smoking. These measures aim to reduce aerosol concentrations and the content of toxic components from the source [174, 175]. In addition, strengthening ambient air quality monitoring, establishing concentration early warning mechanisms, and releasing real‐time information to guide the population to reduce outdoor activities during high‐exposure periods are important supplements to source control.

Population protection should specifically cover high‐exposure groups (e.g., sanitation workers, traffic police, industrial workers) and susceptible populations (e.g., the elderly, children, individuals with genetic susceptibility to PD). High‐exposure groups can wear standard masks such as N95 during work to reduce respiratory inhalation exposure. Susceptible populations should avoid going out during high‐pollution periods, take protective measures when going out, maintain indoor ventilation, and use air purifiers to reduce indoor exposure [176]. Furthermore, health education to improve public awareness of aerosol neurotoxicity, guiding the development of healthy habits such as regular work and rest, a balanced diet, and moderate exercise, can enhance the body's antioxidant capacity and immunity, improve tolerance to toxicity, and serve as an important means of population protection.

#### 4.5.4 Early Warning and Precision Intervention: Screening and Application of Biomarkers

Pathological damage induced by atmospheric aerosols in PD is insidious, initiating decades before the onset of clinical symptoms. Therefore, establishing an early warning system based on biomarkers to achieve early screening and intervention is crucial for improving patient prognosis and reducing incidence. Currently identified relevant biomarkers are mainly classified into three categories: exposure, effect, and diagnostic biomarkers. Their combined application can realize early warning and precision intervention of PD.

Exposure biomarkers are used to assess population aerosol exposure levels, including metal ions (e.g., iron, manganese, copper) and PAH metabolites (e.g., 1‐hydroxypyrene) in blood and urine. Detecting their concentrations can clarify individual exposure dose and duration, screen high‐exposure populations, and provide a basis for targeted protective interventions. Effect biomarkers are used to evaluate early body damage, including oxidative stress indicators (e.g., MDA, SOD, glutathione GSH), inflammatory cytokines (e.g., IL‐1β, TNF‐α, IL‐6), α‐syn oligomers, and microRNAs (miRNAs) such as miR‐7, miR‐133b, and miR‐155. These biomarkers become abnormal before the appearance of PD clinical symptoms, reflecting the process of early neural damage and providing a reference for early warning. Diagnostic biomarkers are used for early PD diagnosis, including α‐syn, homovanillic acid (HVA) in cerebrospinal fluid, and brain imaging biomarkers. Combining diagnostic biomarkers with effect biomarkers can improve the accuracy of early diagnosis.

In the future, with the development of technologies such as multiomics and single‐cell sequencing, it is expected to screen new biomarkers with higher specificity and sensitivity, construct multidimensional early warning models, and achieve precise early screening of aerosol‐related PD [177, 178]. Meanwhile, based on biomarker detection results, personalized intervention strategies can be formulated for populations with different exposure levels and damage degrees, realizing precise prevention and treatment and further improving intervention effects.

### 4.6 Challenges in Current Research and Future Directions

This review collates extensive existing literature to outline atmospheric aerosol toxic components, brain entry pathways, core pathological molecular cascades, research models and potential intervention strategies. However, the initial manuscript merely compiled published results in a descriptive manner, without rigorous critical evaluation of the quality, reliability and limitations of evidence generated from different research categories. A large number of contradictory research findings exist in this field, and the underlying reasons for these inconsistencies have not been fully analyzed and explained.

Significant discrepancies can be observed across epidemiological, toxicological and epigenetic studies. In population investigations, some cohort studies report a linear positive dose–response correlation between long‐term PM2.5 exposure and sporadic PD risk, while other surveys only detect threshold effects or fail to find significant associations. Such conflicts arise from inconsistent exposure quantification methods, incomplete adjustment for confounding factors including age, genetic susceptibility and lifestyle, as well as regional variations in aerosol chemical composition. In toxicological experiments, debates remain regarding the combined toxic effects of mixed aerosol pollutants: transition metals (Mn, Fe, Cu) and PAHs show synergistic neurotoxicity in certain tests, yet partial antagonism is observed under low‐concentration co‐exposure conditions. Research on epigenetics and glial responses also presents divergent outcomes. The expression trends of key microRNAs (miR‐7, miR‐155) and the causal order between A1 reactive astrocyte activation and dopaminergic neuronal damage differ sharply across cell culture, intranasal instillation and whole‐body inhalation animal models, which can be attributed to inconsistent exposure duration, concentration gradients and detection time windows adopted in distinct experiments.

Beyond conflicting experimental outcomes, this review only briefly introduced the advantages of in vitro, in vivo, and human research systems, lacking systematic sorting of their widespread methodological flaws. Single‐cell in vitro models oversimplify complex multicellular interactions within the central nervous system and cannot reproduce the chronic low‐dose exposure scenarios seen in real human environments. Neuron‐glia co‐culture models lack intact olfactory epithelial barriers and complete BBB structures, making it impossible to truly simulate the whole process of aerosol translocation into the brain. Rodent animal models have inherent species gaps in respiratory physiology, dopamine metabolism and olfactory neural circuits, so high‐dose toxicological results from animals cannot be directly extrapolated to sporadic PD in humans. Intranasal instillation deviates from natural human respiratory inhalation modes, while whole‐body inhalation equipment is expensive and difficult to maintain stable long‐term low‐concentration exposure environments. Human population research faces additional constraints: exposure levels are indirectly estimated retrospectively, large‐scale prospective cohorts remain insufficient, and confounding variables cannot be fully excluded, which hinders robust causal inference between aerosol exposure and PD onset. Even emerging technologies such as brain organoids and single‐cell sequencing have prominent bottlenecks, including high experimental costs, immature reconstruction of systemic circulation and olfactory brain‐entry pathways, and incomplete restoration of humanized dopaminergic neuron microenvironments.

The preliminary discussion of research challenges only listed scattered unresolved scientific questions, without hierarchical analysis of core knowledge gaps that restrict the progress of this field. Major unresolved issues include the unclear synergistic toxic regulatory networks formed by multi‐component atmospheric aerosols, incomplete clarification of dynamic crosstalk among oxidative stress, neuroinflammation, mitophagy defects and α‐synuclein aggregation, and the absence of validated early biomarkers capable of reflecting subclinical neuronal damage decades before motor symptoms emerge. Most candidate intervention agents targeting oxidative stress, neuroinflammation and epigenetic modification are still limited to cellular and animal preclinical experiments, lacking large‐scale clinical trials to verify their safety and efficacy. Meanwhile, targeted public health protection strategies for high‐exposure occupational populations and genetically susceptible groups have not been fully established.

Comprehensive analysis of interstudy controversies, methodological defects and layered knowledge gaps throughout the manuscript transforms this work from a simple descriptive summary of existing literature into a critical analytical review. It objectively grades the reliability of evidence derived from in vitro toxicology, animal exposure and human epidemiological studies, clarifies the root causes of conflicting experimental data, and greatly improves the scientific depth, critical thinking and guiding value of this review for subsequent mechanistic exploration, model optimization and translational prevention research on aerosol‐related PD.

## 5 Conclusion

PD is a neurodegenerative disease that seriously threatens the physical and mental health of the middle‐aged and elderly population. Its onset and progression are hypothesized to arise from the combined effects of genetic susceptibility, environmental exposure, and nervous system aging. Among these factors, atmospheric aerosol exposure, as a global environmental issue, represents an important environmental risk factor correlated with sporadic PD across multiple epidemiological investigations. Atmospheric aerosols are complex multiphase mixtures containing key toxic components such as metal particles, POPs, and nano/ultrafine particles. These components appear to access the brain through pathways including direct entry via the olfactory pathway, penetration across the lung‐blood‐brain barrier, and inflammation‐mediated alterations in barrier permeability, and may target midbrain substantia nigra dopaminergic neurons in preclinical systems.

Atmospheric aerosol exposure has been proposed to contribute to the degeneration and necrosis of dopaminergic neurons via core molecular cascades including oxidative stress and mitochondrial dysfunction, neuroinflammation and aberrant microglial activation, disrupted protein homeostasis and abnormal aggregation of α‐syn, and altered epigenetic regulation. Meanwhile, neurotoxic effects may be amplified through the positive feedback loop of oxidative stress‐neuroinflammation, the vicious cycle of mitophagy defects and protein aggregation, as well as the synergistic interactions between aerosol toxicants, genetic susceptibility and age‐related aging framed under the “multiple‐hit hypothesis”; these interconnected pathways collectively may facilitate the onset and progression of PD‐related pathology.

Currently, emerging technologies such as in vitro cell models, in vivo animal models, population studies, multi‐omics, brain organoids, and single‐cell sequencing have provided important support for the research on the neurotoxic mechanisms of aerosol‐induced PD. Based on the core molecular mechanisms, potential intervention drugs such as antioxidants and anti‐inflammatory agents, as well as public health intervention measures including source control and population protection, have been developed, offering new ideas for the prevention and treatment of PD. However, the research still faces challenges such as insufficient mechanism elaboration, limited models, uncertain population study results, and imperfect intervention strategies, which need further optimization and breakthroughs in the future.

In the future, by in‐depth analysis of the synergistic neurotoxic mechanisms of multiple aerosol components and the interactive network of molecular pathways, optimizing research models, conducting high‐quality population studies, developing new intervention methods, and strengthening interdisciplinary cooperation, we can further clarify the association mechanism between atmospheric aerosol exposure and PD, improve the system of early warning, precise prevention, and precise treatment of PD, reduce the incidence of PD and improve the quality of life of patients. At the same time, it will provide a reference for the research on air pollution‐related neurodegenerative diseases and promote the sustainable development of the field of environment and health.

## Author Contributions

**Yitian Gao:** writing – original draft, conceptualization, visualization. **Minxiang Dong:** writing – review and editing, conceptualization. **Chenyang Duan:** writing – original draft. **Xiaohan Zheng:** investigation. **Liming Qu:** writing – review and editing, funding acquisition.

## Ethics Statement

The authors have nothing to report.

## Conflicts of Interest

The authors declare no conflicts of interest.

## Data Availability

The data that supports the findings of this study are available in the Supporting Information of this article. The data availability statement is not applicable to this study. The present study is a systematic review and data availability is not applicable.
